# Generation of exogenous germ cells in the ovaries of sterile *NANOS3*-null beef cattle

**DOI:** 10.1038/srep24983

**Published:** 2016-04-27

**Authors:** Atsushi Ideta, Shiro Yamashita, Marie Seki-Soma, Ryosaku Yamaguchi, Shiori Chiba, Haruna Komaki, Tetsuya Ito, Masato Konishi, Yoshito Aoyagi, Yutaka Sendai

**Affiliations:** 1 Research and Development Group, Zen-noh Embryo Transfer Center, Kamishihoro, Hokkaido 080-1407, Japan; 2 Biological Sciences Section, Central Research Institute for Feed and Livestock of Zen-noh, Tsukuba, Ibaraki 300-4204, Japan; 3 Research and Development Group, Zen-noh Institute of Animal Health, Sakura, Chiba 285-0043, Japan; 4 Pig Breeding Laboratory, Central Research Institute for Feed and Livestock of Zen-noh, Kamishihoro, Hokkaido 080-1406, Japan

## Abstract

Blastocyst complementation (BC) systems have enabled *in vivo* generation of organs from allogeneic pluripotent cells, compensating for an empty germ cell niche in gene knockout (KO) animals. Here, we succeeded in producing chimeric beef cattle (Wagyu) by transferring allogenic germ cells into ovaries using somatic cell nuclear transfer and BC technology. The KO of *NANOS3* (*NANOS3*^−/−^) in Wagyu bovine ovaries produced a complete loss of germ cells. Holstein blastomeres (*NANOS3*^+/+^) were injected into *NANOS3*^−/−^ Wagyu embryos. Subsequently, exogenous germ cells (*NANOS3*^+/+^) were identified in the *NANOS3*^−/−^ ovary. These results clearly indicate that allogeneic germ cells can be generated in recipient germ cell-free gonads using cloning and BC technologies.

Genome editing by homologous recombination is considered to be an important tool for generating gene knockout (KO) livestock models. The ability to edit the genomes of livestock animals could improve productivity or breeding capability for worldwide agricultural purposes. Furthermore, genetic manipulation in cattle has more applications, including large-scale production of various valuable proteins, xenotransplantation and disease modelling^1^. Although gene-targeted livestock have been produced successfully by a combination of homologous gene recombination-based targeting strategy and somatic cell nuclear transfer (SCNT), the efficiency of production is extremely low^2^,^3^,^4^.

Chen *et al.* first reported blastocyst complementation (BC), showing that injection of pluripotent cells (PCs) into recombination-activating gene-2 (*Rag2*)-deficient blastocysts generated chimeric mice in which all mature lymphocytes were derived from the PCs^5^. Recently, functional organs have been generated from PCs *in vivo* by BC in organogenesis-disabled mouse and porcine embryos^6^,^7^,^8^. This method could be useful for the generation of human organs into chimeric animals produced by xenogeneic BC.

The *NANOS* gene family is required for germ cell development in diverse organisms, although the processes regulated vary among species and among different gene homologs. *NANOS3* is found in migrating primordial germ cells (PGCs), and the homozygous deficiency of *NANOS3* in the mouse results in the complete loss of germ cells in both sexes^9^. Targeted disruption of *NANOS3* should deplete germ cells from the gonads of *NANOS3*^−/−^ embryos. Large animals are excellent models for human diseases or for producing valuable proteins^10^,^11^,^12^; however, genome-edited animals can exhibit neonatal death or poor reproduction in adulthood^13^,^14^. We believe that the chimerism of PCs in animals exhibiting neonatal death or poor reproduction caused by specific gene KO and fusion with *NANOS3*^−/−^ embryos should result in the generation of germ cells entirely derived from donor animals with a specific gene KO. Thus, this system would enable the stable mass production of specific gene KO animals by mating if it could produce male and female embryos complemented with allogeneic germ cells from the gene-targeted donor animals. This feasibility study demonstrates that *NANOS3*^−/−^ beef cattle derived from early embryos complemented with dairy cattle PCs could be generated with PGCs entirely derived from allogeneic donors.

## Results

### Gene targeting of bovine *NANOS3*

**Figure 1:**
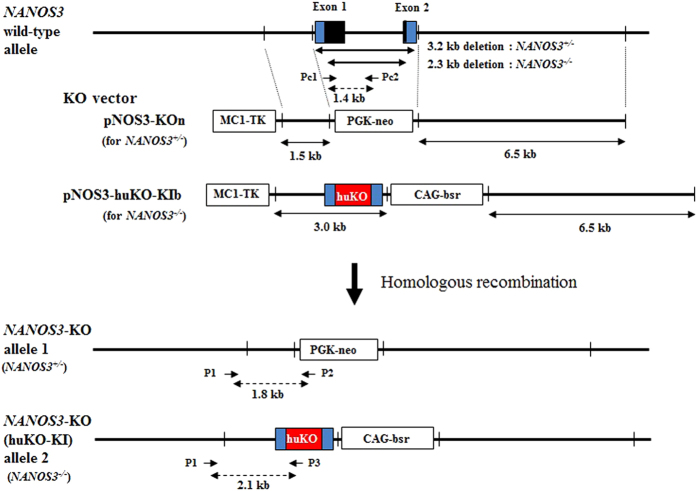
The bovine *NANOS3* gene targeting vector structure, and targeted *NANOS3* KO allele. The coding region is depicted as a closed box (black) and the homologous regions in the targeting vector are indicated by solid lines. The PCR primer pairs (P1 and P2, for targeted allele 1 inserted pNOS3-KOn, 1.8 kb PCR product; P1 and P3, for targeted allele 2 inserted pNOS3-*huKO*-KIb. 2.1 kb PCR product) used to identify *NANOS3* targeted cell clones and KO foetus are indicated by arrows. PCR primers Pc1 and Pc2 (1.4 kb PCR product) were used to identify *NANOS3* deficiency in homozygous KO cells and foetus, and were located in 3.2 kb and 2.3 kb deleted regions generated by homologous recombination.

The bovine vectors targeting *NANOS3* and the gene-targeting scheme are shown in Fig. 1. The vector construct was designed to delete the whole coding region of *NANOS3* by homologous recombination employing an antibiotic resistance gene cassette.

**Figure 2:**
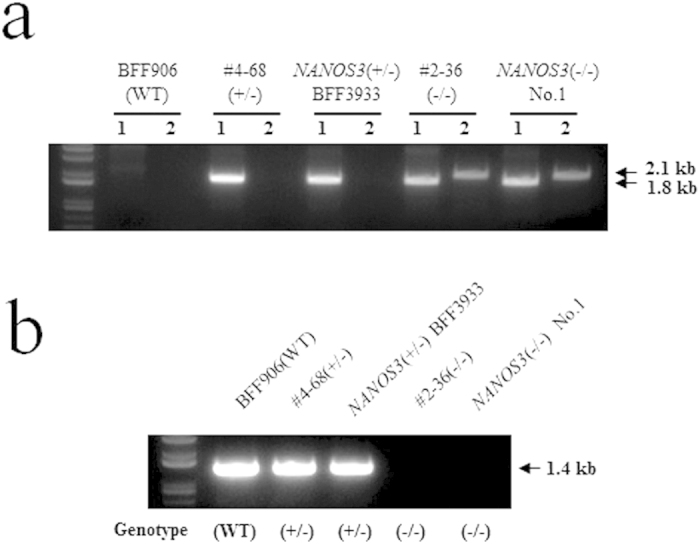
PCR analysis of genomic DNA of *NANOS3* KO BFFs and fetal tissues. (**a**) PCR results for the targeted allele inserted targeting vector transgenes. The lane marked BFF906 displays results from the original BFFs used for preparation of the KO cells. The lanes marked WT in (**a,b**) display results from nontransgenic wild-type controls. Lanes marked #4–68 and *NANOS3* (^+/−^) BFF3933 display results from *NANOS3* heterozygous KO cells and *NANOS3* heterozygous KO BFFs. Lanes marked #2–36 and *NANOS3* (^−/−^) No. 1 display the results from *NANOS3* homozygous KO cells and the uterus of a homozygous KO foetus at day 194 of gestation, respectively. Primers P1, P2 and P3 shown in Fig. 1 were used for PCR analysis. *NANOS3* targeted alleles inserting the targeting vectors pNOS3-KOn and pNOS3-*huKO*-KIb were amplified by PCR using primer pairs P1–P2 (1.8 kb) and P1–P3 (2.1 kb), respectively. Lanes 1 in (**a**) show *NANOS3* targeted allele 1 inserting the targeting vector pNOS3-KOn, and lanes 2 show the *NANOS3* targeted allele 2 inserting the targeting vector pNOS3-*huKO*-KIb. (**b**) PCR results for the deleted region of *NANOS3* gene. WT indicates a nontransgenic wild-type control; *NANOS3* heterozygous and homozygous KO genotypes are shown as (^+/−^) and (^−/−^), respectively. The lane marked BFF906 displays results from the original BFFs used for preparation of the KO cells. Lanes marked #4–68 and *NANOS3* (^+/−^) BFF3933 in both figures display results from *NANOS3* heterozygous KO cells and *NANOS3* heterozygous KO BFFs. Lanes marked #2–36 and *NANOS3* (^−/−^) No. 1 in both figures display the results from *NANOS3* homozygous KO cells and the uterus of homozygous KO foetus at day 194 of gestation. Primers Pc1 and Pc2 (1.4 kb) shown in Fig. 1 were used for PCR analysis. These primers were located in deleted regions generated by homologous recombination.

*NANOS3* targeted cells that underwent homologous recombination were amplified by polymerase chain reaction (PCR) using specific primer pairs (P1 and P2, for the targeted allele 1 inserted pNOS3-KOn, 1.8 kb; P1–P3, for the targeted allele 2 inserted pNOS3-humanized Kusabira-Orange (*huKO*)-KIb, 2.1 kb, dashed line with double-headed arrow shown in Fig. 1), whereas non targeted cells were not (Figs 1 and 2a).

In PCR analysis using the primer pair (Pc1 and Pc2) located at the deleted region of *NANOS3* gene to confirm *NANOS3* deficiency, PCR products (1.4 kb, dashed line with double-headed arrow shown in Fig. 1) were detected in wild-type and *NANOS3* heterozygous KO cells, whereas homozygous KO cells were not (Figs 1 and 2b).

### Production of *NANOS3* ^−/−^ foetuses by SCNT

We established *NANOS3* heterozygous KO cell lines using primary culture cells bovine fetal fibroblast (BFF)906. Nine of the 411 cell colonies obtained were PCR-positive for heterozygous KO vector (pNOS3-KOn) transfection. Among them, four colonies were expanded successfully to confluence in a 75 cm^2^ flask (about 10 days after the PCR checks of the cell colonies) and #4–68 was used for 1st SCNT.

To obtain *NANOS3*^+/−^ BFFs with high proliferative capacity, we produced 43 embryos reconstructed with *NANOS3*^+/−^ cells (#4–68) and the fusion rate of 1st SCNT couplets was 74% (32/43). Seven days after cell fusion, 22% of the embryos (7/32) developed to the blastocyst stage. High-quality blastocysts according to the International Embryo Transfer Society classification^15^ were used for further study. Two of the seven blastocysts were selected and transferred non-surgically into a Holstein heifer to the uterine horn ipsilateral to the existing corpus luteum. Implantation of one of the two embryos transferred to the recipient was observed at day 30. At day 48 of gestation, we retrieved the foetus derived from the *NANOS3*^+/−^ embryo non-surgically. It appeared to be normal morphologically and the size (body length, 35 mm; weight 3.34 g) was similar to that of a previous study^16^. Primary bovine fibroblast cells (BFF3933) derived from the *NANOS3*^+/−^ foetus were established and used for homozygous KO targeting.

To establish *NANOS3* homozygous KO cells using BFF3933 with pNOS3-*huKO*-KIn vector, 15 PCR-positive cell colonies were obtained from the 221 colonies. From the 15 cell colonies, one (#2–36) was selected as *NANOS3*^−/−^ donor cells, and the 2nd SCNT was performed to produce *NANOS3*^−/−^ foetuses. Forty-seven embryos reconstructed with *NANOS3*^−/−^ cells (#2–36) were produced and the fusion rate of 2nd SCNT couplets was 72% (34/47). Seven days after cell fusion, 29% of embryos (10/34) had developed to the blastocyst stage. Two of them were transferred non-surgically into a recipient heifer’s uterine horn. Implantation of one of the embryos was monitored at days 30, 60 and 90. We observed changes in the appearance of the placenta seen by ultrasonography. The foetus derived from the *NANOS3*^−/−^ embryo was retrieved by Caesarean section at day 194 of gestation.

Samples of genomic DNA were extracted from the original BFFs (BFF906), heterozygous KO cells (#4–68, *NANOS3*^+/−^ BFF3933), and homozygous KO cells (#2–36). In addition, uterine tissues were obtained from the *NANOS3*-null foetus (*NANOS3*^−/−^ No. 1) for genomic DNA extraction. The genomic PCR was employed to confirm the presence of the targeted alleles and *NANOS3* deficiency (Fig. 2). The targeted allele with the targeting vector transgenes was detected in the foetus (*NANOS3*^+/−^ BFF3933 and *NANOS3*^−/−^ No. 1), consistent with the donor cells (#4–68 and #2–36) (Fig. 2a). In the analysis of *NANOS3* deficiency, the genomic PCR product of the each foetus was identical to that of the donor cells (Fig. 2b).

**Figure 3:**
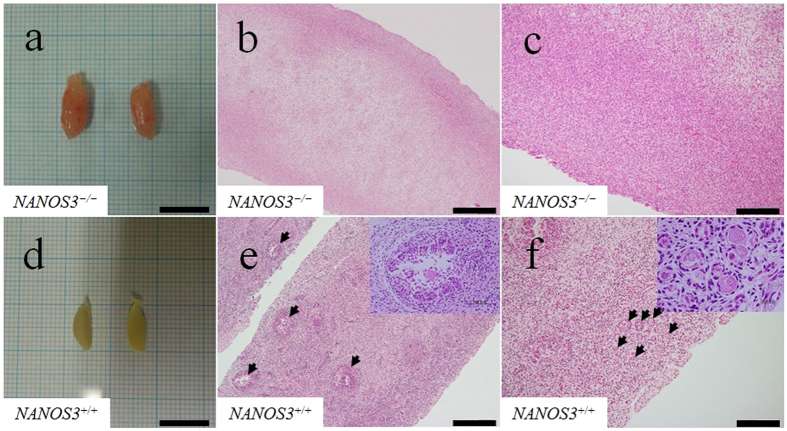
Phenotypes of the *NANOS3* KO ovaries. The morphology and histology of fetal ovaries were compared for *NANOS3*^−/−^ embryos (**a–c**) at 194 days of gestation and *NANOS3*^+/+^ embryos (**d–f**) at 190 days of gestation. No germ cells were observed in the *NANOS3*^−/−^ ovary in (**b,c**). Arrowheads indicate secondary/tertiary follicles (**e**) and primordial follicles (**f**). Scale bars in (**a,d**) = 10 mm; in (**b,e**) = 500 μm; and in (**c,f**) = 200 μm.

The *NANOS3*^−/−^ foetus appeared to be morphologically normal, and its body length (48 cm) and weight (14.0 kg) were almost the same as a wild-type foetus (*NANOS3*^+/+^), derived by artificial insemination (AI), at day 190 of gestation (body length 45 cm; weight 13. 6 kg). The length of the major axis of the *NANOS3*^−/−^ ovaries (11 mm; Fig. 3a) was similar to that of the wild-type ovaries (10 mm, Fig. 3d). Through haematoxylin and eosin (H&E) analysis, no germ cells were observed in the *NANOS3*^−/−^ ovaries (Fig. 3b,c).

### Phenotype and chimerism of chimeric foetuses generated by BC

**Figure 4:**
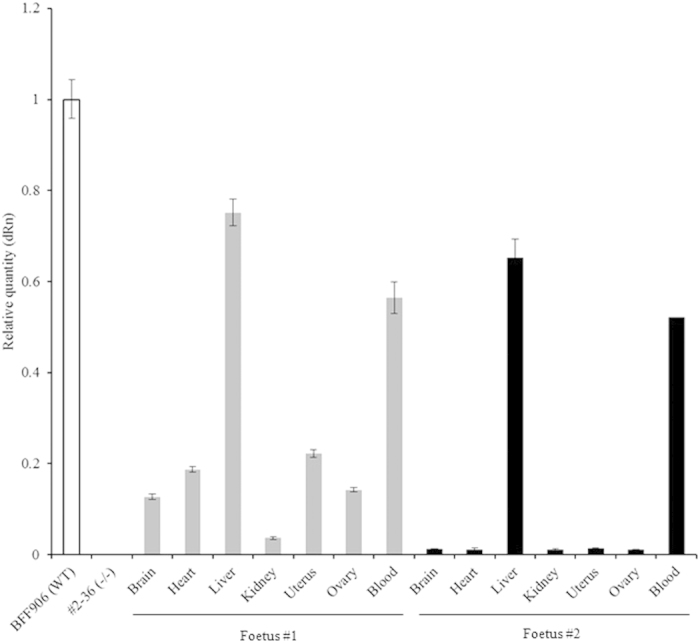
PCR analysis of the genomic DNA of *NANOS3* in chimeric foetuses. Wild-type (BFF906) and homozygous KO [#2–36 (^−/−^)] cell DNA samples were used as positive and negative controls, respectively. Data represent means ± SEMs.

**Figure 5:**
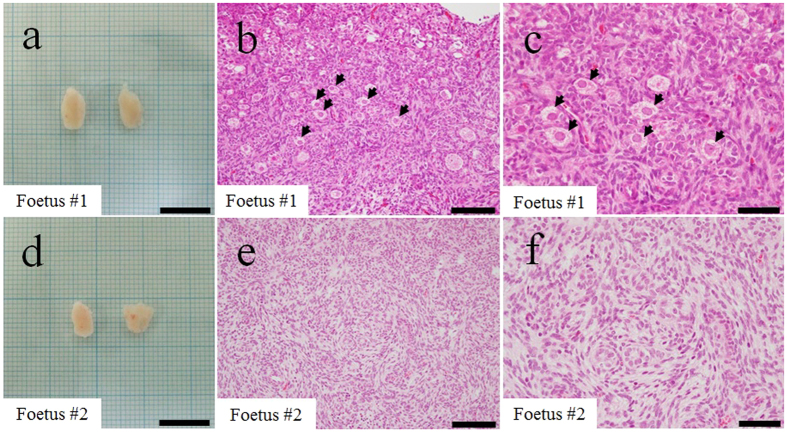
Phenotypes of the chimeric ovaries from *NANOS3*^−/−^ cloned embryos. The morphology and histology of chimeric (#1, **a–c**) and nonchimeric (#2, **d–f**) fetal ovaries were compared. Arrowheads indicate primordial follicles (**b,c**). Although it appeared to be single layer of follicular epithelium-like cells, no germ cells were observed in foetus #2 ovary in (**e,f**). Scale bars in (**a,d**) = 10 mm; in (**b,e**) = 100 μm; and in (**c,f**) = 50 μm.

**Figure 6:**
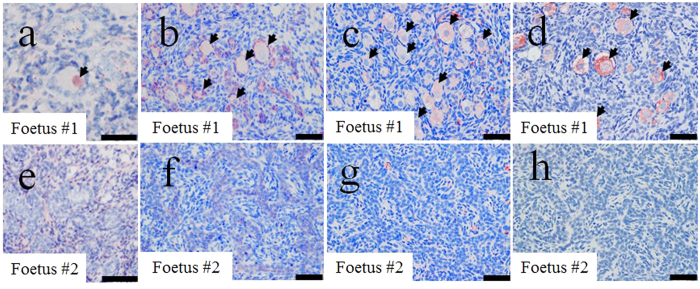
Immunohistochemistry of the chimeric ovaries from *NANOS3*^−/−^ cloned embryos. The histology of chimeric (#1, **a–d**) and nonchimeric (#2, **e–h**) fetal ovaries by ESR1 (**a,e**), NANOS3 (**b,f**), GDF9 (**c,g**) and VASA (**d,h**) immunostaining was compared. Arrowheads indicate primordial follicles (**a–d**). Although it appeared to be single layer of follicular epithelium-like cells, no germ cells were observed in foetus #2 ovary in (**e–h**). Scale bars in (**a–c**,**e–g**) = 50 μm; and in (**d,h**) = 25 μm.

To obtain chimeric bovine foetuses, we produced 87 embryos reconstructed with *NANOS3*^−/−^ cells (#2–36) and the fusion rate of SCNT couplets was 91% (79/87). Five days after cell fusion, 5–10 blastomeres at the morula stage Holstein embryos derived from *in vitro* fertilization (IVF) were injected into the selected *NANOS3*^−/−^ Wagyu embryos (*n* = 17) of the same developmental stage^8^. High quality chimeric blastocysts (*n* = 10) obtained after culture for 2 days were transferred non-surgically into the uterine horns of five recipients (two embryos per recipient). The pregnancy rate of the recipients was 100% (5/5) at day 30 of gestation; however, four of the pregnancies were lost after day 60. Two foetuses derived from chimeric embryos were obtained from one recipient at day 141 of gestation by Caesarean section. They also appeared to be morphologically normal, and their body lengths (foetus #1, 28 cm; #2, 28 cm), weights (foetus #1: 2.0 kg, #2: 2.4 kg) and weights of ovaries (foetus #1: 1.8 g, #2: 1.2 g) were within normal ranges. Chimerism of major organs and tissues (brain, heart, liver, kidney, uterus, ovary and blood) in the two foetuses was substantiated by detecting the *NANOS3* sequence using PCR analysis. The *NANOS3* gene was detected in all tissues of foetus #1 (Fig. 4). However, in foetus #2, it was detected only in the liver and blood cells (Fig. 4). H&E staining (Fig. 5b, c) and estrogen receptor 1 immunostaining (ESR1, Fig. 6a) confirmed the presence of oocytes in ovaries of *NANOS3*^−/−^ foetus. Further immunohistochemical analysis with NANOS3 (Fig. 6b), growth differentiation factor 9 (GDF9, Fig. 6c) and VASA (Fig. 6d) antibodies revealed that oocytes as well as granulosa cells resulted from chimerism between chimeric and non-chimeric foetuses.

## Discussion

*NANOS3* acts in germ cell development in various animals, including flies^17^, frogs^18^, mice^9^, and humans^19^; however, it has not been investigated so far in cattle. *NANOS3*-null mice show infertility resulting from apoptosis of migrating PGCs during the fetal stage, resulting in gonads lacking germ cells^9^. Here we found no apparent abnormalities in the ovaries of *NANOS3*-null cattle that lacking germ cells. Thus the disruption of *NANOS3* also blocks bovine germ cell development. To our knowledge, this is the first report of the generation of bovine species with germ cell deficiency.

We next focused on the generation of exogenous PGCs in the *NANOS3*^−/−^ cloned Wagyu embryos using BC methods. Real-time qPCR analysis (Fig. 4), H&E-stained (Fig. 5) and ESR1, NANOS3, GDF9 and VASA-immunestained (Fig. 6) sections revealed the existence of incorporated exogenous oocytes proper, as well as the granulosa cells of primordial follicles in an ovary on day 141 of pregnancy. Recently, several reports become available, providing the evidence that mixed chimerism can accurately be assessed through the use of real-time qPCR^20^,^21^,^22^. Using real-time qPCR, therefore, we examined whether or not *NANOS3*^+*/*+^ cells derived from the exogenous cells could be identified in major organs and blood of chimeric foetuses. NANOS3 expression is high in human germ cells^19^ and GDF9 and VASA are well-known as markers of the germ cell lineage. Thus, these results confirm the production of chimeric livestock complemented with allogeneic germ cells. This is the first report showing successful complementation of allogeneic germ cells into the ovaries of recipient animals of a different breed in cattle.

This system has various potential applications. One example could be germ cell production from gene-edited animals that not only exhibit poor reproduction, but also show embryonic death caused by specific gene KO. The functional analysis of such germ cells will greatly contribute to the fields of reproductive and developmental biology. Another example would be the generation of xenogeneic germ cells in interspecies chimeras. The establishment of such a system—for example the generation of bovine germ cells in sheep or goats—will help produce valuable bovine germ cells and embryos for the livestock industry. However, KO of the *DAZL* gene also produces male and female sterility and the phenotype shows a loss of germ cells as meiosis starts^23^. Some have reported that the transplantation of wild-type germ cells into *DAZL*-null efferent ductules resulted in the generation of spermatozoa with the donor’s genotype^24^,^25^. However, these approaches have been indispensable for busulfan-treated recipients. Although busulfan is an alkylating agent that is cytotoxic to haematopoietic stem cells and is used to increase the effectiveness of allogeneic germ cell transplantation^26^,^27^, it disrupts spermatogenesis in mammals. Thus, the major advantage of our method using cloning and BC is the avoidance of the need to use busulfan.

Based on our results, there are several issues that need to be examined to validate the functionality of complemented germ cells. We need to obtain full-term calves derived from the same line of chimeric embryos. After birth and growth of the calves, we will perform more analyses of the chimeric animals’ ovary size, follicular development, lineage and fertility of the oocytes, embryonic development, and implantation capacity following AI. In this study, two foetuses (#1, a chimeric foetus and #2, a nonchimeric foetus) derived from chimeric embryos were obtained from a recipient heifer. The weights of ovaries from foetus #1 (1.8 g) were 1.5–fold heavier than those of foetus #2 (1.2 g). This result may reflect successful complementation of alien germ cells in the ovary of only foetus #1. In foetus #2, the *NANOS3* gene was detected only in the liver and blood cells, with a chimerism level of approximately 50–65% (Fig. 4). This indicated that blood chimerism could have occurred by the exchange of blood cells between bovine foetuses through vascular anastomoses between the placentas in early pregnancy, as seen in freemartinism. Accordingly, we suggest that the chimerism of the liver in foetus #2 arose in a similar manner. In the fully chimeric foetus #1, allogeneic cells were found in the ovary and all other major organs and tissues. The chimerism levels of the *NANOS3* gene ranged from 4% (kidney) to 75% (liver) (Fig. 4). It has been reported that the generation rate of exogenous liver in KO mice is higher than that of kidney from pluripotent cells via BC^7^,^28^. These results might be the reason for uneven distribution of exogenous cells in various organs. A few studies have considered the generation of human organs into chimeric animals produced by xenogeneic BC^6^,^8^; however, the generation of human PGCs in other animal species will raise major ethical problems. To avoid this problem, KO of the *NANOS3* gene could become a powerful tool, in that the deficiency of the *NANOS3* gene in donor human PCs might suppress the differentiation of human PGCs in the gonads of other animals.

In conclusion, the generation of foreign germ cells in ovaries derived from a *NANOS3*-null cow was performed successfully. Our finding will accelerate the utilization of genome-edited large animals for biomedical and agricultural purposes. Furthermore, induction of biallelic mutations by homozygous KO should save considerable time in the production of null mutant livestock. Recently, Cong *et al.*^29^ and Mali *et al.*^30^ reported that the CRISPR/Cas9 systems are technically simple and effective methods for gene disruption, allowing generation of large animals with specific gene KO by direct zygote injection. Combining the CRISPR/Cas9 system and our developed technology would greatly enhance the commercial potential of using these techniques in KO animal production.

## Methods

### Ethics statement and animal care

All experiments were performed in accordance with the Guidelines for the Husbandry and Management of Laboratory Animals of Zen-noh ET Center, and approved by the Zen-noh ET Center Animal Experiment Committee and Biosafety Committee. Recipient heifers (14**–**18 months old) were all fed the same diet, and water was supplied *ad libitum*. The herds were comprised considering body constitution and social hierarchy.

### Chemicals

All chemicals were purchased from Sigma-Aldrich (St. Louis, MO, USA) unless otherwise stated.

### Preparation of BFFs

Primary cultures of BFFs were obtained from a 49-day-old Wagyu female foetus (BFF906). Briefly, the cells were cultured in DMEM (Invitrogen, Carlsbad, CA, USA) containing 10% (v/v) FBS (Japan Bio Serum, Hiroshima, Japan), 2 mM l-glutamine and 1 mM sodium pyruvate for one passage at 38.5 °C under 5% CO_2_ in air with saturated humidity.

### Construction of *NANOS3* KO vectors and isolation of *NANOS3* KO cells

Gene targeting of bovine *NANOS3* was performed as described^1^,^31^. The bovine *NANOS3* gene-targeting vector was designed using the bovine *NANOS3* gene sequence (Accession Number NC007305.5, region 10061880) in the National Center for Biotechnology Information database. For construction of the *NANOS3* KO vector, the short homologous arm (1.5 kb fragment) located at 5′ outside of the *NANOS3* gene was generated by PCR using the forward primer 5′–CTCTCCGTTGCATCCATGCC–3′ and the reverse primer 5′–AGCCACTGACCTTCCAGCTGAC–3′ and DNA of Wagyu BFFs. Likewise, the long homologous arm (6.5 kb fragment) located at 3′ outside of the *NANOS3* gene was generated using the forward primer, 5′–GGACAAGGTATCGTGAACTGC–3′ and the reverse primer, 5′–AACACGAGGAGCACCTTCTTGC–3′. The *NANOS3* heterozygous KO vector (pNOS3-KOn) was constructed by insertion of the homologous arms into the phosphoglycerate kinase I promoter with neomycin resistance gene (PGK-neo)/MC1-TK plasmid vector (Fig. 1). For construction of the homozygous KO vector (pNOS3-*huKO*-KIb), a 2.0 kb fragment containing a part of the *NANOS3* Exon 1, and a 0.35 kb fragment containing a part of the *NANOS3* Exon 2 were generated using primer pair I (forward 5′–CTCTCCGTTGCATCCATGCC-3′ and reverse 5′–AGGTTGCTCGAGCCCATGGCTGG–3′) and primer pair II (forward 5′–CAATTGCTTCTGCCTAAGGAGACTGG–3′ and reverse 5′–CAATTGGCAGTTCACGATACCTTGTCC–3′), respectively. Then, the 3.0 kb short homologous arm of the *NANOS3* coding region was replaced with the 0.66 kb *huKO* gene constructed using 2.0 kb and 0.35 kb fragments. The KO vector pNOS3-*huKO*-KIb was constructed by the insertion of the 3.0 kb short and the 6.5 kb long arms into the CAG–blasticidin resistance gene (*bsr*)/MC1-TK plasmid vector (Fig. 1). The preparation of bovine *NANOS3* KO cells was performed as described^1^. Briefly, transfection of the *NANOS3* KO vector pNOS3-KOn was performed using electroporation. BFFs (1 × 10^7^ cells) were transfected with 5 μg of pNOS3-KOn vector at 220 V and 950 μF using a Gene Pulser apparatus (Bio-Rad Laboratories, Hercules, CA, USA). Transfected cells were cultured in 10% FBS in Minimum Essential Medium α (Invitrogen) in a 6-well plate for 48 h. After incubation, the transfected cells were suspended with 400 μg/ml geneticin (Invitrogen) and 20 μM ganciclovir (Nacalai Tesque, Inc., Kyoto, Japan) for positive/negative selection, respectively. For preparation of the *NANOS3* homozygous KO cells, *NANOS3* heterozygous KO BFFs (*NANOS3*^+/−^ BFF) were obtained from a *NANOS3* heterozygous Wagyu foetus produced by SCNT. These cells were then transfected with the KO vector pNOS3-*huKO*-KIb using the method described above. For selection of homozygous KO cells, 10 μg/ml blasticidin (Invitrogen), 400 μg/ml geneticin and 20 μM ganciclovir were added to the selected heterozygous KO cells. After being cultured for 10 days, each cell colony was separated into two parts and then cultured continuously. After 24–48 h, one of the colonies was isolated and used for PCR analysis (Fig. 2). Positive KO cells were grown to confluence in a 75 cm^2^ flask (Nunc, Nalge Nunc International, Roskilde, Denmark) and cryopreserved until SCNT.

### Oocyte maturation and SCNT

Thirty to 40 cumulus–oocyte complexes (COCs) were cultured for 20 h in 700 μl of tissue culture medium 199 containing 25 mM N-2-hydroxyethylpoperazine-N′-2-ethane sulfonic acid (Invitrogen) and 5% (v/v) FBS in 4-well tissue culture plates (Nunc) under sterile paraffin oil at 38.5 °C under 5% CO_2_ in air with saturated humidity. After maturation in culture, oocyte enucleation was achieved by removal of the first polar body and metaphase II plate in a small volume of cytoplasm using micromanipulation^32^. The G0/G1 phase cells were drawn into a 15 μm (inside diameter) glass pipette (Microtech IVF, Brno, Czech Republic) and quickly inserted into the perivitelline space of the enucleated oocytes. After 24 h of maturation of the oocytes, the donor cell–recipient cytoplast couplets were fused by an electric DC pulse. The SCNT-generated embryos were activated using a conventional method^32^.

### IVF procedure

*In vitro* matured oocytes from a Holstein cow were inseminated with sexed Holstein semen (CRI Genex Cooperative, Inc., Shawano, WI, USA). Briefly, motile spermatozoa were obtained by discontinuous density gradient centrifugation of frozen-thawed bull spermatozoa in 90/45% (v/v) Percoll. Mature COCs were then co-cultured with the Percoll-separated spermatozoa (2.5 × 10^6^ spermatozoa/ml) for 6 h as described^33^.

### *In vitro* culture of embryos and generation of chimeric embryos

SCNT- or IVF-derived embryos were cultured in 700 μl of CR1aa medium^34^ with 5% (v/v) FBS (FBS-CR1aa) in 4-well tissue culture plates for 5–7 days at 38.5 °C in 5% O_2_, 5% CO_2_ and 90% N_2_ with saturated humidity. IVF-derived embryos at day 5 were sexed with a Loop-mediated Isothermal Amplification Kit (Eiken Chemical, Tokyo, Japan), and 5–10 female blastomeres were drawn into a 30 μm (inside diameter) glass pipette (Microtech IVF) and carefully inserted into the perivitelline space of *NANOS3*^−/−^ cloned embryos at day 5^8^. The chimeric embryos were cultured further in FBS-CR1aa medium for 2 days.

### Embryo transfer (ET) to recipient heifers and recovery of bovine foetuses

Bovine embryos were loaded into clear plastic straws (0.25 cm^3^) and transferred nonsurgically into Holstein heifers (two embryos per recipient) to the uterine horn ipsilateral to the existing corpus luteum using an ET device (YT GUN, Yamane-teq Co., Ltd., Nagano, Japan) on days 6–8 of the oestrous cycle (day of oestrus = day 0). Pregnancy was determined by real-time B-mode ultrasonography (Convex scanner HS–1500, Honda Electronics Co. Ltd., Toyohashi, Japan). Transcervical recovery of foetuses at around day 50 was carried out according to the modified protocol of Ideta *et al.*^16^. Briefly, luteolysis was induced by two intramuscular (i.m.) injections of 0.5 mg cloprostenol (Resipron®-C, 0.25 mg/ml, ASKA Pharmaceutical Co., Ltd., Tokyo, Japan) given 4 h apart. At 36 h after the first cloprostenol treatment, several treatments of 25 mg dinoprost (Veterinary Pronalgon®-F Injection, 5 mg/ml, Pfizer Animal Health, Tokyo, Japan) were introduced into the cervix 1 or 2 h apart using a sheath (Fujihira Industry Co., Tokyo, Japan) for AI. Oxytocin (100 IU, Atonin®-O, ASKA Pharmaceutical Co.) was administered i.m. when protrusion of the fetal membranes from the vulva or movement of the foetus towards the cervix was detected by ultrasonography. Bovine foetuses at mid- to late-gestation were obtained by Caesarean section^35^.

### Evaluation of *NANOS3* deficiency

The original BFFs, *NANOS3*^+/−^ heterozygous KO cells, and *NANOS3*^−/−^ homozygous KO cells were examined by genomic PCR analysis to validate KO efficiency. Genomic DNA extraction and PCRs were conducted using FlexiGene DNA kits (Qiagen, Venlo, the Netherlands) and Prime STAR GXL DNA Polymerase (Takara Bio Inc., Otsu, Japan) according to the manufacturers’ protocols. The primers used to detect the targeted allele 1 inserting pNOS3-KOn vector were: P1, 5′–AACACGGTGAAGCTCACTTAGG–3′ and P2, 5′–GATGCTCCAGACTGCCTTGG–3′. The PCR conditions were: 95 °C for 1 min, 57 °C for 1 min, and 72 °C for 2 min for 35 cycles. The primers used to detect the targeted allele 2 inserting pNOS3-*huKO*-KIb vector were: P1 (above) and P3, 5′–CTTCATCTCGGGCTTGATCGTCG–3′. The primer P1is located at the outside of 1.5 kb short homologous arm genome region (shown in Fig. 1). The primers P2 and P3 are located at the PGK promoter and *huKO* regions, respectively. The primers used to confirm *NANOS3* deficiency were: Pc1, 5′–CCACGTGCTCAAGGATGAAGC–3′ and Pc2, 5′–CTGATACGTAAGCCTAGCTACTCG–3′. The primers Pc1 and Pc2 were located in deleted regions generated by homologous recombination (shown in Fig. 1). The PCR cycle conditions were the same as above. After PCR, a 15 μl aliquot of each product was loaded on to a 1% agarose gel, separated electrophoretically, and the ethidium bromide-stained gel was photographed^1^.

### Detection of chimerism by real-time quantitative (q) PCR

The major organs and blood of chimeric foetuses (*n* = 2) were examined by real-time qPCR analysis to identify *NANOS3*^+/+^ cells derived from the transplanted Holstein blastomeres. Genomic DNA extraction was conducted using DNeasy Blood & Tissue kits (Qiagen) for organs and Nucleo Spin Blood (Machery-Nagel, Düren, Germany) for blood samples. To validate the real-time qPCR chimerism analysis, reactions were performed in triplicate (*n* = 2) in a volume of 20 μl with Brilliant III Ultra-Fast SYBR Green QPCR Master Mix (Agilent Technologies, Santa Clara, CA, USA) on an Mx3000P thermal cycler (Agilent Technologies). The qPCR conditions were: preincubation for 3 min at 95 °C, 40 cycles of 20 s at 95 °C, and 20 s at 60 °C. Following real-time PCR, melting curve assays were performed to identify specific amplification products. The primers used to detect donor *NANOS3*^+/+^ cells were forward 5′–CTGGAGGCTCCCTAAGGTCT–3′ and reverse 5′–CTAGCTACTCGAAGCCTGCC–3′. The gene for basic transcription factor 3 (*BTF3*) was used as a reference for qPCR experiments as reported previously to determine the bovine gene copy number^36^. The primers used to detect *BTF3* were forward 5′–AGCAGAAGCAGAAGTCTAAGCA–3′ and reverse 5′–GGGAGATTACTAAGGGTAAAGCGA–3′. Standard amplification curves were plotted for donor *NANOS3*^+/+^ cells. DNA samples were made with over six serially halved dilutions and the amplification efficiency for each primer set was calculated using MxPro QPCR software (Agilent Technologies).

### Ovarian histology and immunostaining

Ovaries from the wild-type (day 190 of gestation), *NANOS3*^−/−^ cloned foetus (day 194 of gestation) and chimeric (day 141 of gestation) foetuses were fixed immediately in 4% neutral buffered formalin (Wako Pure Chemical Industries, Ltd., Osaka, Japan) for 24 h for histological analysis. The fixed ovaries were dehydrated through a graded series of ethanol, embedded in paraffin wax, serially sectioned at 5 μm, and mounted onto clean glass slides. Sections were dewaxed in xylene and rehydrated through a decreasing series of ethanol concentrations. For each specimen, at least three to five sections were stained with H&E. Further, steroids play an activity role in the growth, differentiation, and function of ovarian follicles in cattle. Activity of bovine germ cells was detected immunohistochemically for an anti-ESR1 antibody (21244-1-AP, 1:50 dilution; Proteintech, Chicago, IL, USA) as previously described^37^. Bovine follicle formation occurs during fetal life. Follicles were classified as described by Fair *et al.*^38^ with modification. To indicate the evidence of allogenic contribution in the *NANOS3*^−/−^ ovary, the alien germ cells were immunostained with anti-NANOS3 antibody (1:100 dilution; kindly donated by Dr. Y. Saga)^39^. The transforming growth factor beta superfamily *GDF9* and *VASA*, also called *DXD4* (DEAD [Asp–Glu–Ala–Asp] box polypeptide 4) or *MVH* (mouse *VASA*–homologue), are known as markers of the germ cell lineage in cattle. The allogenic germ cells in *NANOS3*^−/−^ ovary were immunostained with anti-GDF9 (ab93892, 1:50 dilution; Abcam, Cambridge, MA, USA) and VASA (ab13840, 1:50 dilution; Abcam) antibodies^40^.

## Additional Information

**How to cite this article**: Ideta, A. *et al.* Generation of exogenous germ cells in the ovaries of sterile *NANOS3*-null beef cattle. *Sci. Rep.* **6**, 24983; doi: 10.1038/srep24983 (2016).
